# Supplementary material for: Associations between objective and self-perceived physical activity and participation in everyday activities in mild stroke survivors
Source: PLoS One. 2025 Apr 1;20(4):e0321047. doi: 10.1371/journal.pone.0321047 (PMC11960890; doi:10.1371/journal.pone.0321047)
Supplement: Attachment — Submitted filename: Thank you for the opportunity to review the manuscript.docx [file pone.0321047.s002.docx]

Thank you for the opportunity to review the manuscript, ‘Association between objective and self-perceived levels of physical activity and participation in daily life activities in mild stroke survivors. Though it is an interesting article there are numerous methodological issues. The following references can improve the quality of the manuscript.

1. Line 49 state mild stroke survivors. Only mild stroke survivors were included in the study?

2. Was the choice of International Physical Activity Questionnaire (IPAQ-SF) based on its relevance?

3.Line No. 137-138 Statement” The IPAQ has demonstrated satisfactory psychometric properties in stroke survivors” The authors are encouraged to explain in the manuscript.

4. Line 50 in abstract and Line no 275 – 278 in discussion states different things. Please clarify? 6. Could authors clarify on which basis quantitative variables were expressed as mean and standard deviations and/or median depending on the data distribution. As not mention about any test of Normality used or not.

7. In statistical analysis section, line 215- 217, please provide an explanation for using Spearman rank correlation (Non parametric test) why and why not Parametric test (Pearson correlation coefficient test).

8. Could authors Clarify why not using multiple regression/ logistic regression to find out association or relationship between PA and participation in daily life activities in Spanish mild stroke survivors.
